# Picture Your Nursing Home: Exploring the Sense of Home of Older Residents through Photography

**DOI:** 10.1155/2015/312931

**Published:** 2015-08-05

**Authors:** J. van Hoof, M. M. Verhagen, E. J. M. Wouters, H. R. Marston, M. D. Rijnaard, B. M. Janssen

**Affiliations:** ^1^Centre for Healthcare and Technology (Fontys EGT), Fontys University of Applied Sciences, Dominee Theodor Fliednerstraat 2, 5631 BN Eindhoven, Netherlands; ^2^Institute of Allied Health Professions, Fontys University of Applied Sciences, Dominee Theodor Fliednerstraat 2, 5631 BN Eindhoven, Netherlands; ^3^Section of Applied Gerontology, Institute of Nursing, Fontys University of Applied Sciences, Dominee Theodor Fliednerstraat 2, 5631 BN Eindhoven, Netherlands; ^4^Centre for Research Computing, Faculty of Mathematics, Computing and Technology, Open University, Milton Keynes, Buckinghamshire, MK6 7AA, UK

## Abstract

The quality of the built environment can impact the quality of life and the sense of home of nursing home residents. This study investigated (1) which factors in the physical and social environment correlate with the sense of home of the residents and (2) which environmental factors are most meaningful. Twelve participants engaged in a qualitative study, in which photography was as a supportive tool for subsequent interviews. The data were analysed based on the six phases by Braun and Clarke. The four themes identified are (1) the physical view; (2) mobility and accessibility; (3) space, place, and personal belongings; and (4) the social environment and activities. A holistic understanding of which features of the built environment are appreciated by the residents can lead to the design and retrofitting of nursing homes that are more in line with personal wishes.

## 1. Introduction

For older people who can no longer age in place, nursing homes provide an alternative place of residence where care and assistance are offered by professionals. The needs and wishes of older residents in terms of the architectural quality of the nursing home environment should ideally be respected and implemented as much as possible. It is questionable whether the nursing home is a place where one feels at home which corresponds to how residents experience the environment in which they reside. Furthermore, such questions correspond to what is needed for residents to feel at home in the first instance, which, in turn, should be considered in the constant process of evaluation and action. Creating attractive nursing homes, from both setting and social perspective, in which residents can feel at home, is a core challenge of nursing home organisations, in particular, their care staff and facility managers [1–4]. Too often, existing nursing homes do not meet the needs of the current generations of residents, and this mismatch leads to suboptimal living conditions that are experienced 24 hours a day.

In order to create this form of environment, namely, a true nursing “home,” it is essential to address the experiences and views of the residents in studies and empower the thoughts of these residents through a combination of inclusive design strategies [5, 6]. Despite the laudability of small-scale initiatives in healthcare [7, 8], (future) residents are often overlooked and not included in design and development processes.

In the second half of the 20th century, the design of nursing homes was conducted by experts in the field, and this isolated approach traditionally resulted in high-rise hospital-like buildings [2]. In such cases, the quality of the building was often expressed in terms of technological, functional, and economic requirements, and factors related to the residents were given less attention. A more holistic vision of healthcare is currently emerging which considers the consequences of the built environment on the well-being of the residents [2]. For instance, Van Steenwinkel et al. [2] conducted a study focusing on how the built environment contributes to a feeling of homeliness of older people living in different contexts in Belgium. Van Dijck-Heinen et al. [3] investigated the sense of home among permanent and temporary residents of nursing homes in the Netherlands. Both studies concluded that a sense of home is a multifactorial phenomenon which is highly influenced by environmental, social, and personal characteristics. When one experiences a positive sense of home, one might experience a feeling of familiarity [9]. Duyvendak [10] also uses the two metaphors “heaven” and “haven” to describe a sense of home in a better way. Heaven refers to a place where you can be yourself, feel connected with like-minded people, and perform your favourite activities. Haven is a safe, comfortable, predictable place. When referring to the characteristics of “haven,” this is primarily associated with the physical environment [10]. On the one hand, there is an elaborate set of literatures related to heaven and how social interactions with significant others play an important role in experiencing a sense of home. The literature related to haven, on the other hand, which deals with the built environment and personal belongings, is less abundant [4]. Despite these research efforts, there is a gap in the literature with respect to what might “influence” a sense of home and the general well-being of, and according to, nursing home residents with physical limitations and gerontopsychiatric health problems.

Therefore, this study explores (1) which factors in the physical and social environment correlate with the sense of home of the residents and (2) which environmental factors are most meaningful. The goal of this study is to gain insight into the experiences and views of actual residents in order to understand their needs in relation to the design of nursing homes and in order to make these nursing homes more fitting and facilitating to the social context of residents. This study is part of a larger programme called “Nursing Home of the Future,” which investigates the architectural, technological, and social aspects of future nursing homes and the sense of home of the residents [1, 11–13].

## 2. Methodology

A qualitative methodology was chosen for this study, comprising photography and in-depth interviews with nursing home residents, as a way to involve the least voiced people in our society through creativity and dialogue [5]. The Critical Appraisal Skills Programme's checklist for qualitative research [14] was used as a guide for this study. In the following sections we describe (1) the settings, ethics, and participants, (2) photography approach, (3) interviews, and (4) the data analysis.

### 2.1. Settings, Ethics, and Participants

In April and May 2014, interviews and the field study were conducted in two wards of the same nursing home in Eindhoven, the Netherlands, including 12 participants (Table 1). The study aimed to include both residents with physical limitations and gerontopsychiatric health problems in order to gain a more diverse set of results. The inclusion criteria for this research were as follows: participants had to be at least 55 years of age, had to reside in a nursing home for at least six months, had to be able to communicate in Dutch, had to be able to take pictures with a photo camera independently or with the help of (in)formal carers, had to be able to make a selection of important photos independently, and had to be able to participate in an interview of at least 30 minutes.

A total of nine residents with physical limitations (out of 25 who were asked to participate) and three additional gerontopsychiatric residents (who were selected by the care organisation) consented to their participation. Prospective participants and their relatives received an information letter from the principal care professional, which was approved by the hosting care organisation. Informed consent was obtained from the participants in conjunction with their initial family carers by signing the given consent forms. All documentation was treated anonymously. Moreover, no persons or images of persons were included in these photos in a recognisable way. Participants were asked not to take explicit pictures or photographs of people who did not want to be photographed.

The personal data from the participants were obtained with a checklist, which was based on the Tilburg Frailty Indicator (TFI) [15], an instrument used to quantify the frailty of older people from their own perspective. The study population consisted of 8 females and 4 males. The age of the participants ranged from 62 to 95 years. Eight of the participants were widowed, two respondents were married, and two were unmarried. All participants were born in the Netherlands. The level of education varied from primary education only (*n* = 5) to secondary (*n* = 4) and higher education (*n* = 3). Eight participants indicated, using the TFI, they felt healthy, three participants had no clear indication of their overall health, and one participant indicated not feeling healthy. The duration of residence in the nursing home ranged from 8 months to 8 years. Every participant had a single-person room, with private sanitary facilities. The participants had stated that they were satisfied with their living environment.

### 2.2. Photography

As pointed out by Annemans et al. [16], we experience the built environment through our senses. Therefore, a visual research method was chosen for this study [17], in which people are interviewed based on photographs they have taken themselves with cameras supplied to them by the researcher. Radley [17] concluded that what pictures portray and what stories narrate are better thought of as versions of our experience of the world than as constructions of the world that we experience.

Photography is a method that has been used in research for decades and which allows participants to create a record of an event, capture a complex phenomenon, or tell a story through images [18–21]. As taking photos does not rely on language alone, it can be used with vulnerable populations who might not normally be included in research [18, 22], for example, in researching frail older persons and nursing home residents. Previously, photography methods have been applied in the domains of housing, communities, and the built environment [16, 23–28].

Images provide a lasting record of an event or in the case of this study an architectural or social scene, required to study the relationship of humans and their living environments. A photo only serves as a conduit to enhanced, thoughtful, and deliberative narrative, instead of as a replacement of words. Photography allows complex environmental, health, and social issues to be captured and then shared with other people [16, 18]. There are numerous photography methodologies available for researchers, including photovoice, photo-elicitation, and photo-production methods. As this study tries to capture the real-life experiences of nursing home residents, the photo-production method was applied, which builds on the principles of photo-elicitation [17]. In line with Annemans et al. [16], this study wanted to gain a good understanding of what has been made visible on the photos, in addition to why and how. The photo-production method enables researchers to experience a phenomenon from another point of view, in this study, the nursing home as a living environment. The older residents, who serve as participants, are experts of their own situation and environment, and they can highlight the positive and negative features in the built environment.

Studies by other researchers, for instance, Yuan and Dong [28], suggest that asking older people to make pictures to document their experiences is not always straightforward. In this study, the selection procedure, the instructions, and manual were tools to tackle potential operational challenges.

At the beginning of this study, the participants received a short explanation of the goal of the study and how to use the camera, including a leaflet-sized written and visual manual on how to use the camera. The manual also contained the study's time plan, its goals, and contact details of the principal researcher (M. M. Verhagen). The participants were asked to share their experiences and views concerning their living environment and which of the factors of the living environment were most and least meaningful, through taking photographs of characteristic elements and situations.

The research team chose to provide the participants with disposable cameras instead of digital cameras for three reasons. First, traditional cameras may be more familiar to the participants, as is stated by Novek et al. [29], and can be operated as long as residents have sufficient strength and dexterity in their hands. Second, disposable cameras are cheaper than digital cameras and when dropped are not as easily damaged. Third, using disposable cameras limits the number of pictures participants could take, limiting the choice in the selection procedure. A disadvantage of disposable cameras is that pictures need to be developed, which is time consuming and costs money and one does not know the end results immediately. Digital cameras yield digital output, which is easier to process for data analysis. Moreover, participants may be less cautious with the disposable camera as they know it is of a lower value. With both methods, pictures can be supplied to the participants after the research as a token of appreciation of their contribution to the research.

In this study, participants were supplied with a camera for one week up to a week and a half (in late April and early May 2014) and were asked to take pictures of their living environment. The reel of the camera contained 27 photos, which should be sufficient for taking the most important pictures, but participants were free in the number of pictures they took. The participants did not keep a log of the things they photographed, although the researchers were aware that due to privacy restrictions not every situation could be photographed [30]. Even though notes from the logs could be used during the elicitation process, we assumed that the limited number of pictures would not require a log. Support, for instance, by family carers, could be provided to the participants for taking the photographs, but the decision to take a picture and the choice for scene that was to be photographed had to be that of the participant. After the time period, the cameras were collected by the principal investigator and sent off for development of the photos. Upon completion, the principal investigator returned to the participants and invited them to choose a top-5 ranking of their photographs. The top-5 ranking was used as a basis for the interviews held by the principal investigator in May 2014, as participants could use the selection as a foundation for what they wanted to discuss in the interview [31].

### 2.3. Interviews

During the subsequent photo-elicitation interviewing phase, participants talked about the photographs and how they attributed social and personal meanings and values to these photographs. All interviews were conducted within the private rooms of the residents. The interviews had the character of a conversation in which participants were asked about their experiences and views of their living environment and their sense of home. The interviews varied between 30 and 60 minutes each, depending on the richness of the conversation and the attention span of the participants.

Each interview commenced with an introduction and participants were asked about their personal background, the reasons for admission, and health status (Table 1). The opening question included whether participants could describe their experiences concerning their living environment in general. Thereafter, the top-5 photographs taken were discussed in terms of the contents of the photographic material. This resulted in participants being asked to describe why the particular pictures were selected and the meaning of the pictures to the individual. The top-5 ranking was supplemented by items from a topic list, which is an overview of research themes and accompanying questions. The list was based on the work of Van Steenwinkel et al. [2] and van Dijck-Heinen et al. [3]. The participants were free to add items to the topic list, as long as these were relevant to the study. The topic list contained items relating to the living environment, such as (1) appreciation (choice, most meaningful features), (2) the choice for the top 5 (choice, meaning, positive features, and improvements), (3) experiences, wishes, and expectations of the living environment, (4) experience of the sense of home (which experiences, positive experiences, and improvements), and (5) experiences in relation to the living environment (which experiences, positive experiences, and improvements).

### 2.4. Data Analysis

**Figure 1:**
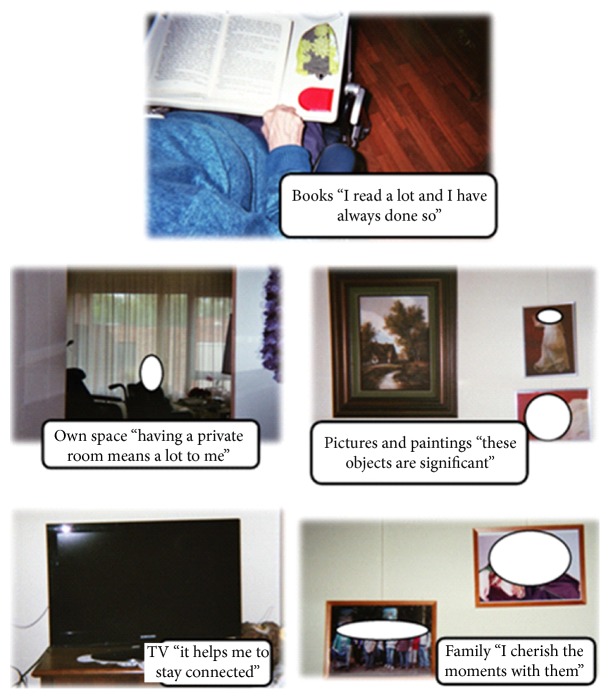
Example of the top-5 pictures of one of the participants, showing personal belongings, a private room, and social relationships. The top-5 ranking does not necessarily show building-related photos only.

All interviews were recorded with a voice recorder and transcribed verbatim. The data were analysed based on the six phases by Braun and Clarke [32]. First, all transcripts were read in their entirety. Then, the top-5 photographs were compared to the transcripts (Figure 1), which were used in conjunction with the photographs, and were evaluated in terms of the values that are attributed to the statements made by the participants and the photographs they had taken.

For instance, were these statements either positive or negative? Then, the first set of codes was being generated through open coding. Thereafter, codes were added to the transcripts. The researchers, bearing the research questions in mind, systematically highlighted the relevant information (open coding). Open coding concerns the process of unravelling all of the collected data into fragments or codes. Similar codes and quotes were clustered and labelled, and themes emerged from this process. Together, the research team organised the codes and clustered them into smaller thematic groups. The final themes were grouped in amalgamation with the photographs that reflected aspects of these themes, which is a form of axial coding. Thereafter, these themes were reviewed and then defined and named. In order to guarantee the anonymity and privacy of participants, the photographs showing people were processed in order to white out faces. Names of people and institutions, appearing in quotes, were deleted from the written texts or were changed.

## 3. Results

There are numerous factors and subfactors which influence the sense of home. The thematic analysis led to the identification of four themes: (1) the physical view; (2) mobility and accessibility; (3) space, place, and personal belongings; and (4) the social environment and activities. These themes are further elaborated in the following sections.

### 3.1. The Physical View

The theme named the physical view encompasses the green environment in which the nursing home is located and the views from the building. Every participant stated that they appreciated the green environment of the nursing home, which is located in a park landscape. Living amongst nature is considered a positive perspective. A number of residents stated that the environment is beautiful, healthy, and green. This does not mean that all residents can appreciate the landscape by going outside, but in such cases the view of nature is considered to be attractive and pretty. To some, the green environment provides a sense of freedom. In total, the green environment was placed four times at the top 5 of photographs by the residents and the view from the nursing home five times. Three out of twelve participants mentioned the green environment. Words association with the green environment included “enjoying,” “healthy air,” “freedom,” and “nice and green.”

* A green environment. Yes, it is healthy and beautiful, isn't it? […] It is so nice, well, you need to enjoy it, I can really enjoy it. I always say that it is the small things that matter. That is the way it is, and you need to appreciate that. (Participant 5)*

Seven participants mentioned the view. Word association with the view included “beautiful,” “nice,” and “freedom.” One of the participants talked about the photo she took staring out of the window, which she experienced as freedom.

* I always look outside from the window over there. Freedom, aren't I allowed to say that? (Participant 1)*

Participants did not make a distinction between the types of view from the room. Any type of view was appreciated in this study, whether it was a park, traffic, a playground with children, or a building. The main reason for appreciating the view was that it gives them something to look at during daytime.

* It is beautiful view, over this piece of art, the water tower. (Participant 9)*

A regular feature was the importance of birds. At one of the living rooms in the gerontopsychiatric ward there was a wild crow, eating from a net of peanuts hanging in front of the window. One of the respondents stated that this bird was the main source of distraction and a “resident of the ward” as if it were a pet animal.

### 3.2. Mobility

Several participants spoke of elements which can be classified within the theme of mobility, which relate to freedom, independence, and interior design. One of the residents spoke of the elevator, which is hard to operate when seated in a wheelchair. The hoist was mentioned as being an essential element in the room in order to be comfortable and to aid getting out of the chair. The bathroom can be a source of joy, if people can access it without difficulties, and they can wash and shower themselves. Another participant spoke of the washbasin in her room, which is hard to use, and therefore, it was photographed.

* Yes, this one is about the sanitary equipment. You need to have a decent washbasin … one of those washbasins for the disabled, so to say. So you can wash your hands without having to stretch to absurd proportions. (Participant 8)*

*A little difficult, isn't it? It, of course, does not feel like living at home, but on the other side it is pleasant to have someone around the corner when you need to go to the bathroom, so to say. (Participant 6)*

Two participants indicated that the taxi service was important in order to travel to places such as the supermarket and to see relatives. One of the participants stated that the waiting times are often too long, although they should be around 30 minutes at the most.

*I'm happy that there is a taxi service. It opens the way to somewhere else. But things don't go smoothly. (Participant 9)*

Participant 7 showed a picture of him being lifted out of the bed. The hoist was named “James,” in analogy to a butler, but the respondent said there was nothing interesting about the technology itself. No special meaning was given to the hoist, other than that it supported the personal mobility of the participant.

In relation to mobility, being dependent and the lack of freedom were the least appreciated features in the lives of the residents. One of the participants mentioned their wish to be able to walk again, because it would improve their sense of being independent. One of the respondents mentioned the feeling of not being able to do things alone and thought that this aspect was the worst of living in a nursing home. One of the female residents took a picture of her diseased hands (arthritis) and described them as a source of “sadness.” Another resident stated that having to sit all day makes one feel dependent on others, because one has to ask others to do things for them, and another one felt locked-up occasionally.

*The nursing home, it is something you have to experience yourself for once. Having to sit here all day, being dependent, having to ask for everything. It is hard to cope. (Participant 4)*

*Well, I cannot say that I always enjoy myself, but that is more a matter of my character than of the environment I think. […] Yes, I do feel locked-up once in a while. (Participant 7)*

One participant stated that he had lost the sense of freedom because he was no longer allowed to drive around in a mobility scooter, which also emerged in the theme of mobility.

*Yes, because of the mobility scooter that was taken away from me, I lost a great part of my freedom. (Participant 7)*

### 3.3. Space, Place, and Personal Belongings

Many photographs were taken by participants based on pieces of furniture or personal belongings that are present in their room. The theme also concerns being able to decorate their own room and deciding which personal items are important in this process. Three of the participants took photographs of their television set. These residents cannot think of having to abandon the television, as it helps them get through the day and to keep up to date with the world news. This is actually one of the few comments about technology by the residents.

A regular feature was the importance of flowers. Flowers contribute to a homely atmosphere, as was acknowledged by multiple participants. Drawings of grandchildren meant a lot to the participants, because they represented memories of loved ones. One of the residents explicitly stated personal belongings and having a nice chair are important to her.

*Yes here, the fact that I have a comfortable chair and many of my personal things. That is something I like. (Participant 6)*

Having access to either a single-person room or an allocated space in the communal areas is subthemed as having a private place in this study. This theme emerged from the analysis of the photographs. Five participants took photographs of their own private place. This was either their own room (4 participants) or a place somewhere else in the nursing home. Additional pictures were taken of picture frames with smaller photos of the relatives, which were found in the private rooms. During meal times, the residents have their own place around the table. The private room means pleasure and joy in living. One of the participants wished that their own room would have a more attractive atmosphere but has the impression that personal belongings and furniture would not fit inside. She does not know what the possibilities are for bringing items, and she does not want to burden others with having to carry loads.

*I wish the room were bit cozier, but there is nothing at home that would fit in here. I have large cabinets, and I have a large clock too. It would be nice if I could put it here, but I don't bother to carry heavy loads. And a small cabinet as well, and a large one, but I am not sure whether it will stand in the way. We need space for the patient hoist and it would not be enough [with the cabinet]. (Participant 3)*

Two participants stated that living in the nursing home will never be like living at home but that they have accepted the situation and are satisfied. Two other participants stated that they do not experience a sense of home at all. One of the residents stated it feels like being in a shared student dormitory.

*Do I have to give a grade for it? Well, I don't have a sense of home. It feels like I live in a student house, we are going back to our teenage years, living in a room and getting pocket money. (Participant 5)*

For one of the participants, the common living room is the main source of a sense of home. Two of the participants stated that they would rather return to their old home. They did realise that this was no longer an option. Four of the participants stated that the way they are being cared for positively contributes to a sense of home. Two residents experienced a sense of home inside of their private rooms.

*Because I am rather mobile, and have my own stuff around, I do feel at home in my room. (Participant 6)*

One participant mentioned the entrance as being important. She calls the entrance her private spot and forms a sense of mental freedom. Residents may have a sense of being locked.

*The entrance is important. Because it is also the exit. (Participant 5)*

*Without this little place, I don't make it through the day. There is a lot of buzz. (Participant 10)*

Finally, one of the participants mentioned the cleaning tools and products in her room. The meaning of these items was a sense that the room was kept clean and tidy. She was worried that the current cleaning regimen of cleaning twice a week was reduced to once a week, which she considered to be insufficient.

### 3.4. Social Environment and Activities

The theme named social environment and activities encompasses the contacts with care professional and relatives and being engaged in activities. The participants indicated a great appreciation for the care staff; in particular, the personal contact and the treatment were considered positive. A number of participants indicated that they valued their contact with relatives as most positive, in particular, having close contacts and being visited on a regular basis.

A number of times, participants spoke of their interaction with care professionals. Five out of twelve participants stated that these professionals contributed positively to their quality of life. One of the participants took a picture of herself with a care professional. It was perceived important to have a positive personal contact with the caregiver and being able to get along well with them.

*Well, yes, the contact with the care professionals. Well, yes, you need to be on speaking terms, so we can get along well. (Participant 9)*

Five of the participants mentioned the importance of relatives during the interviews and included spouses, children, grandchildren, great grandchildren, parents, and siblings. One of the participants mentioned that he appreciated that his wife came to visit him every day at 18:00.

*The fact that my wife comes visit me every day is the thing I appreciate most. (Participant 9)*

A number of participants spoke about the activities that were being organised, such as the cooking club, arts, and crafts such as paint workshops, and reminiscence activities conducted in the activity space. One of the residents spoke of the small shop on the ground floor which keeps her occupied and allows her to buy clothes. Two others indicated missing a large store in the direct neighbourhood for buying groceries.

*Oh that is so nice. I had to go to the small store a while ago. If I cannot buy the things I want, they will actually buy it for me. (Participant 10)*

Having dinner is an ingredient for employing a nice atmosphere to multiple participants. One of the participants took a photograph of a Chinese meal, which he found illustrative of the atmosphere and the memory he had of the dinner. Another participant took a photo of a quality restaurant and the quality food that was served there. She said she missed good quality food in the nursing home. In the two wards of the nursing home, food and drinks were experienced differently. Food, to the participants, is related to appreciation of the living environment which is a positive or negative sense.

*Sometimes, the food is alright, and at other times it is very bad. I have the choice between two meals, and I guess I always chose the wrong meal. (Participant 7)*

*I appreciate the food the most. It looks so tasty and it actually tastes good. People told me I was getting served wartime meals; well these meals were definitely not served during the war. It is all rubbish what they said. (Participant 10)*

A number of residents valued the dining atmosphere, especially in relation to being seated around a table together.

*Particularly going to the dining room is nice. We all sit together around a table with certain people. The table is nicely set. You sit around the table like you used to do at home. (Participant 2)*

## 4. Discussion and Conclusion

### 4.1. Reflections on the Results

The focus of this study was the sense of home. This is a complicated and multifactorial phenomenon, which contributes to a sense of identity and well-being of nursing home residents [3, 33–35]. The themes which seem to have a relationship with the experienced sense of home in this study seem consistent with the findings of Sixsmith [36]. In this study, 22 adults were questioned about which factors were associated with their experienced sense of home. 20 categories were formed which were categorised into three layers of perceiving a home, namely, physical, social, and personal.

Feeling at home is a layered emotion [10], and in order to experience a sense of home, one should feel familiar within the environment. A home can either be a country, a city, a neighbourhood, a house or a park bench [9], or, in the context of this study, a nursing home. This, however, does not mean that it is a naturalness that nursing home residents experience a sense of home by just the fact that they live in such a facility. Not all of the residents in this study felt at home, even though they could point out factors in the environment that were important to them. The feeling, that is, the sense of home, that we are talking about is a secondary emotion, and this is a conscious experience of the experienced “more reflexive” primary emotions [37]. Additional emotions play a role, which can differ per individual and one's circumstances. Our study showed that certain social events, including having meals together with fellow residents, contribute to positive emotions that, in turn, positively contribute to their personal sense of home.

A well-experienced balance between autonomy and safety/security also contributes to a positive sense of home [2, 38]. In this study, residents express concerns and limitations related to their sense of autonomy, for instance, when speaking of the need for assistance and help with mobility. At the same time, the sense of security is also hampered in the nursing home, as people are dependent on others for help. In the words of these residents, their sense of home is affected in a negative fashion. This can be explained through the nature of nursing homes, in which more emphasis is put on the provision of a safe and secure environment, whereas residents like to see an environment in which their personal autonomy is enhanced. If a person does not experience a good sense of home, he or she may feel anxious and uncertain [9], and this, in turn, may be aggravated by the fact that the sense of autonomy and security in a nursing home environment may be lower than that for people who still live in their own homes. Duyvendak [39] also reported that people can feel homesick and nostalgic when they experience a lack of sense of home. One's own home can be seen as a secure place from which they can explore the world around themselves [2]. By furnishing one's home with personal belongings, for instance, one enables reminiscence and positive memories [2, 34, 40, 41]. In this study, residents mentioned the importance of personal belongings as a way to connect with their past and as a source of familiarity.

### 4.2. There Is More to It Than Just the Built Environment

When architects design buildings, it is often thought that the built environment and the architectural design dictate how people experience the space and the interactions that will take place. To residents, the built environment may be of a different level of importance compared to the more basic needs and a well-functioning and stimulating social environment. This does not rule out the need for age-friendly solutions and accessibility. Primarily, residents value good communication with care professionals and relatives the most, as well as having a sense of autonomy, independence, and freedom. These are all human values. This is in line with findings from a review by Xu et al. [42] on the quality of life in relation to nursing home features. The researchers concluded that there were serious questions about whether any elements of the nursing home's structure can improve residents' quality of life. Rural facilities (green environments) and facilities with a higher percentage of private rooms had better residents' self-reported quality of life. This is in line with the statements by the residents that they valued a green environment. Moreover, it suggests that the built environment has a role to play in the quality of life of older people but that the social environment should also be considered when planning and designing a new facility.

According to Brown et al. [24], place attachments are the positive bonds that people form with places and which arise from affective, behavioural, and cognitive ties between individuals or groups and their sociophysical settings. The built environment cannot be seen on its own without regarding the social context. Moreover, place attachments can also change, as old attachments evolve or are disrupted and new attachments form [24]. Admission to a nursing home is a major life-event, as most individuals do not wish to leave the home they have been living in for a long time in order to move to a nursing home [43]. Nevertheless, “*there seem to be good reasons to assert that living in an institution and being ‘at home' is not a contradiction in terms*” [44, page 221]. In this study, residents spoke of (social) activities that took place in a certain room or part of the nursing home, for instance, eating in the communal living room. These activities and spaces were mentioned at the same time, as if they were linked together. The activities were the main contributor to the development of a sense of home and may be a starting point to improve living conditions for people who have been admitted to a nursing home.

Still, features of the built environment have a role to play. Features of the built environment that matter are the green environment, the view from the rooms, and having personal belongings in a private room where residents can withdraw. When these important features of a nursing home are present, residents have a basis to develop a sense of home. Moreover, these features are within reach of architects designing nursing home facilities. It is, therefore, an important task for architects to create environments which meet the needs of future residents and may even contribute to social interaction, for example, by designing rooms where one can meet up and interact with others and where visitors are invited and feel welcome. The need for having both social and architectural components which have to be fulfilled in order to get a sense of home matches the findings by Van Steenwinkel et al. [2] of people who resided in the community. In our study, participants indicated that having flower arrangements and having a positive and homely atmosphere and habits, like eating together, are important for experiencing a sense of home. These statements are in line with earlier findings by Cooney [45].

What this study has shown once more is that nursing home residents are independent and unique in their needs and wishes. It is important not to offer standardised arrangements for nursing homes. Instead, residents should have the freedom to live the lives they want to lead and make changes to their room (by adding personal belongings) without hampering the provision of care. People in general express their sense of identity through their personal belongings [10, 46], and these belongings can “move” the sense of home to the nursing home environment [47]. Relatives can play a role in the development of a sense of home in the nursing home, for instance, in eating together, decorating their room, saying goodbye to their former home, and the creation of a new home together with other residents. The photographs and outcomes of the interviews may actually act as the stimulus for social action and change [48]. In line with such actions, it should be interesting to explore the degree of freedom a nursing home resident has in relation to making modifications to the home environment or private room.

### 4.3. Reflections on the Methodology

The methodology applied in this study can be utilised across a wide range of settings, including older people with dementia, with autism, and with cognitive impairments. People have a strong visual tool to express wishes and needs, when words are often too limited to describe how people think or feel about something. In fact, the methodology could also be expanded to indoor environmental research and studies concerning building services engineering at home and in office buildings. The photographs allow for a direct and complete expression of thoughts and expressions and provide a qualitative methodology which is not entirely new to the field [16, 28, 49] but is not applied on a large scale in nursing homes. There are some things that can be improved in future studies concerning nursing home residents.

First of all, the researcher could undertake the photography in conjunction with the participants and conduct the interviews directly afterwards or even during the photography sessions. The participants will be able to remember why a certain picture was taken in the first instance and provide a better description. This is particularly true when it takes some weeks to have the photographs developed (as traditional photography is getting out of fashion and is no longer optimally supported by the market chain). Using a digital camera is more costly but solves the challenge of having to wait. In addition, using disposable cameras resulted in half of the photographs being blurred and, therefore, not useful for the study. When dealing with poorly taken photographs, one could ask for making top 5 without the photographs but by describing what has been photographed in order to find answers to the research question. When taking photographs together with the participants, one can do a secondary check with the participants by again showing the photographs and the transcripts in a later stage as a form of member check. Given the small sample size of the current study, it would be recommended to work with larger groups. This would also allow for the application of the so-called photovoice methodology [17] in which themes can be found through interactions in a group.

### 4.4. Conclusions

The major challenge for practice is how to incorporate the factors identified in this study in the design and planning of the built environment and how to stimulate social interactions in order to improve the sense of home of residents. The residents' sense of home is not similar to the design of housing alone. The implications thereof are that improving a sense of home is a multifactorial assignment in which many factors need to be addressed and that it encompasses more than just a good design of the built environment. This is also reflected by the statements of the residents included in this study, as a sense of home was not experienced by all of them. The treatment by others, including relatives and care professionals, is another important factor that is valued and has an impact on the sense of home.

The improvement of the experienced sense of home can be achieved through the residents themselves on the one hand and the care professionals on the other hand, for instance, by making changes to the built environment and social interactions. An inclusive approach to the design of nursing homes can combine creative and interviewing techniques which help create an ideal home situation. It can provide a way of participation for the least voiced. In the future, nursing home organisations and family carers could engage in similar creative methods in order to find out which aspects of the built environment are appreciated most and how rooms should be designed and decorated. This would be a method to stimulate social change which is inclusive.

## Figures and Tables

**Table 1:** Characteristics of the participants.

Participant	Sex [M/F]	Age	Marital status	Education	Limitations
1	M	84	Widowed	Primary education	Mobility
2	M	94	Widowed	Secondary education	Mobility, early dementia
3	F	85	Widowed	Secondary education	Mobility
4	F	88	Widowed	Primary education	Mobility
5	F	74	Widowed	Secondary education	Mobility, CVA
6	F	87	Widowed	Primary education	Mobility, hearing
7	M	95	Widowed	Higher education	Mobility, sight
8	F	64	Unmarried	Higher education	Mobility
9	M	78	Married	Higher education	Mobility, CVA
10	F	83	Widowed	Primary education	Mobility, psychiatric symptoms
11	F	62	Unmarried	Primary education	Mental/intellectual problems
12	F	67	Married	Secondary education	Mobility, psychiatric symptoms
